# MSTCN: A multiscale temporal convolutional network for user independent human activity recognition

**DOI:** 10.12688/f1000research.73175.1

**Published:** 2021-12-08

**Authors:** Sarmela Raja Sekaran, Ying Han Pang, Goh Fan Ling, Ooi Shih Yin

**Affiliations:** 1 Faculty of Information Science and Technology, Multimedia University, Ayer Keroh, Melaka, 75450, Malaysia; 2 Millapp Sdn Bhd, Bangsar South, Kuala Lumpur, 59200, Malaysia

**Keywords:** human activity recognition, smartphone, temporal convolutional network, dilated convolution, one-dimensional inertial sensor

## Abstract

**Background:** In recent years, human activity recognition (HAR) has been an active research topic due to its widespread application in various fields such as healthcare, sports, patient monitoring, etc. HAR approaches can be categorised as handcrafted feature methods (HCF) and deep learning methods (DL). HCF involves complex data pre-processing and manual feature extraction in which the models may be exposed to high bias and crucial implicit pattern loss. Hence, DL approaches are introduced due to their exceptional recognition performance. Convolutional Neural Network (CNN) extracts spatial features while preserving localisation. However, it hardly captures temporal features. Recurrent Neural Network (RNN) learns temporal features, but it is susceptible to gradient vanishing and suffers from short-term memory problems. Unlike RNN, Long-Short Term Memory network has a relatively longer-term dependency. However, it consumes higher computation and memory because it computes and stores partial results at each level.

**Methods:** This work proposes a novel multiscale temporal convolutional network (MSTCN) based on the Inception model with a temporal convolutional architecture. Unlike HCF methods, MSTCN requires minimal pre-processing and no manual feature engineering. Further, multiple separable convolutions with different-sized kernels are used in MSTCN for multiscale feature extraction. Dilations are applied to each separable convolution to enlarge the receptive fields without increasing the model parameters. Moreover, residual connections are utilised to prevent information loss and gradient vanishing. These features enable MSTCN to possess a longer effective history while maintaining a relatively low in-network computation.

**Results:** The performance of MSTCN is evaluated on UCI and WISDM datasets using subject independent protocol with no overlapping subjects between the training and testing sets. MSTCN achieves F1 scores of 0.9752 on UCI and 0.9470 on WISDM.

**Conclusion:** The proposed MSTCN dominates the other state-of-the-art methods by acquiring high recognition accuracies without requiring any manual feature engineering.

## Introduction

Human activity recognition (HAR) is extensively applied in various applications such as personal health monitoring, ^1, 2^ geriatric patient monitoring, ^3^ ambient assisted living, ^4^ etc. The widespread use of smartphone-based HAR is due to the ubiquity of smartphones and low-cost sensors. Additionally, sensor-based HAR provides a non-intrusive solution.

Numerous HAR algorithms have been proposed, including handcrafted feature (HCF) methods ^5- 7^ and deep learning (DL) methods. ^8- 10^ HCF methods require complex data pre-processing and manual feature engineering. The manually extracted features are highly dependent on prior knowledge, leading to high bias and loss of essential implicit patterns. Hence, DL methods, such as convolutional neural network (CNN), ^8, 9^ recurrent neural network (RNN), and long-short term memory network (LSTM), ^10, 11^ are devised to overcome the downfalls of HCF methods. DL methods involve no complex data pre-processing, and features are automatically tuned for the desired outcome. Besides, the architecture is adaptable to different applications.

Although CNN is good in extracting spatial features, it hardly learns temporal features, which are significant in motion analysis. RNN and LSTM are feasible for time-series data, but they suffer from several shortcomings. For example, RNN is prone to short-term memory problems, leaving out important information at the beginning if the input sequence is too long. LSTM prevails over RNN as the former has a longer-term dependency and is less susceptible to vanishing gradient. However, LSTM requires higher computation due to multiple gate operations and more memory to store partial results throughout the training phase.

**Figure 1.**
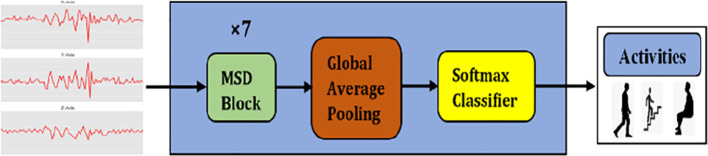
Architecture of MSTCN.

This work proposes a multiscale temporal convolutional network, termed MSTCN. As illustrated in Figure 1, MSTCN is constituted by multiscale dilation (MSD) blocks, global average pooling and softmax. The contributions of this work are:

- A deep analytic model, amalgamating the Inception model and Temporal Convolutional Network (TCN), is developed to extract spatial-temporal features from inertial data. MSTCN requires minimal data pre-processing and no manual feature engineering.
- Multiple different-sized convolutions are incorporated in MSTCN to perform multiscale feature extraction. The scaled features encompass low-to-high level features of the data. The concatenation of multiscale features enables MSTCN for better data generalisation.
- Dilated convolution is implemented to improve the convolution kernel's receptive fields. The dilation captures the global characteristics of the inertial data and retains a longer effective history.
- A comprehensive experimental analysis is conducted using two popular public databases, UCI ^12^ and WISDM. ^13^ Subject independent protocol is implemented where the training and testing sets do not share the data from the same users.

### Related work

One-dimensional inertial data undergoes a complicated pre-processing in HCF methods to extract salient statistical feature vectors in time and/or frequency domains. The manually extracted features are then fed into standard machine learning classifiers, such as support vector machine (SVM), ^7, 12^ ADA Boost, ^14^ Random Forest, ^6^ C4.5 decision tree, ^15^ etc., for activity classification. He and Jin ^5^ proposed a discrete cosine transform method to extract features and classify the features using multiclass SVM. Lara *et al*., ^16^ developed an additive logistic regression, boosting with an ensemble of 10 decision stump classifiers. In the works of Ronao and Cho, ^17, 18^ the authors explored the Continuous Hidden Markov Model (HMM) to perform activity recognition in two stages, where the first stage is for static and dynamic classification and the second stage is for course classification. Although these methods produce an adequate performance, they are highly dependent on the effectiveness of the manual feature engineering techniques.

Recently, researchers leaned towards DL methods since DL requires minimal to zero pre-processing and feature engineering. Ronao *et al*., ^8^ Yazdanbakhsh *et al*., ^9^ and Huang *et al*., ^18^ proposed a CNN-based deep learning system to perform HAR. The reported empirical results show the feasibility of the CNN-based method in analysing motion data. Besides, three-layer LSTM was proposed to classify human activities. ^11^ LSTM variant, known as Bidirectional LSTM, was employed in HAR. ^10^ This model uses richer information, i.e. previous and subsequent information, to perform activity recognition. Nair *et al*. proposed two variations of TCN, namely Dilated-TCN and Encoder-Decoder TCN in HAR. ^19^ In addition, another two TCN-based models are proposed in Ref. 20, namely TCN-FullyConnectedNetwork and deepConvTCN. Both works of Nair *et al*., ^19^ and Garcia *et al*., ^20^ concluded that the TCN-based models achieved better performance than existing recurrent models due to their longer-term dependencies.

## Methods and results

**Figure 2.**
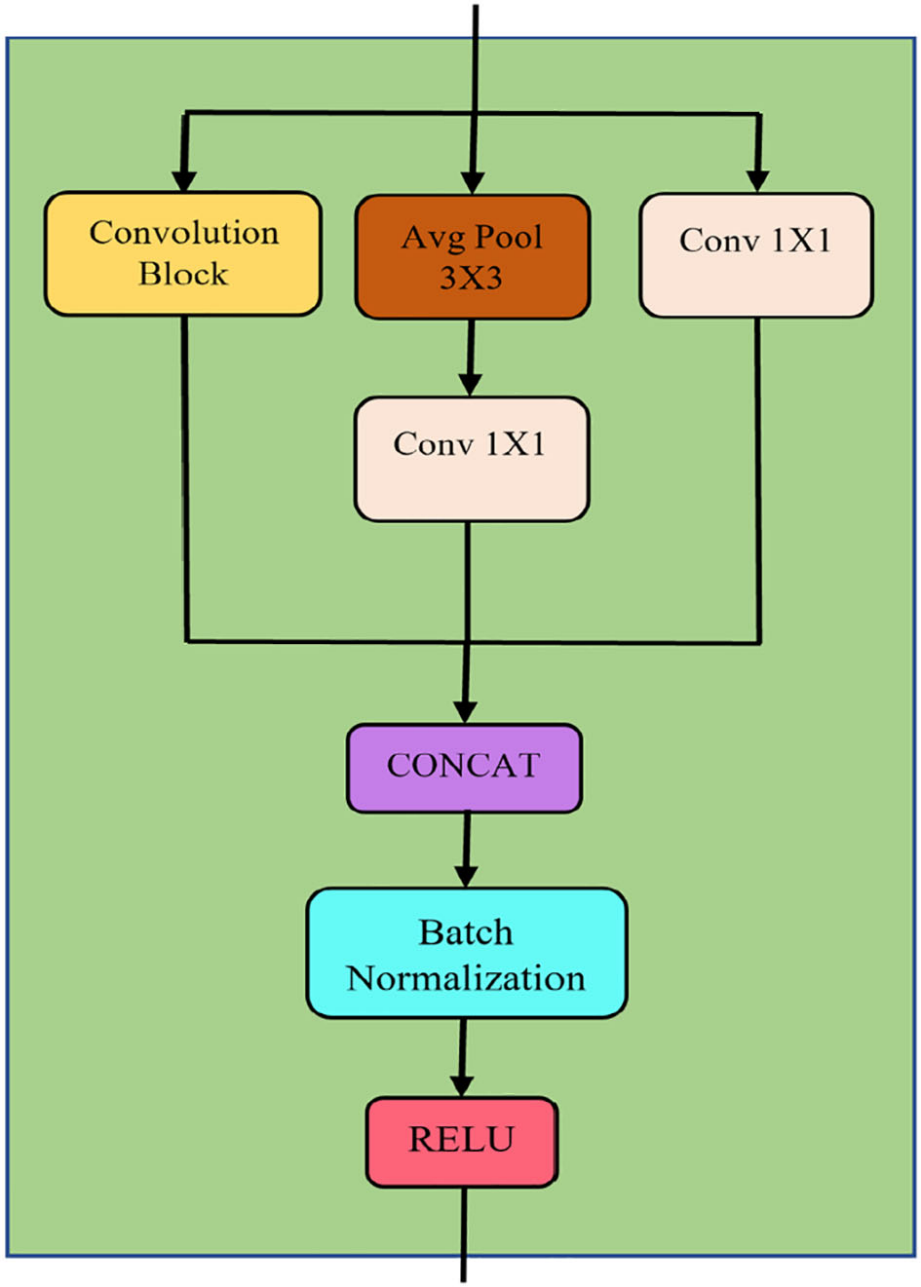
MSD block.

The raw inertial signals were first pre-processed to remove any null values. Next, the pre-processed signals were segmented using sliding window technique. In specific, the signals were partitioned into fixed-sized time windows and each window did not intersect with another window. Then, the segmented data was fed into seven MSD blocks in MSTCN (green box in Figure 1) for feature extraction. Figure 2 illustrates the structure of an MSD block. MSD block was designed based on the inception module structure for multiple scale feature extraction. ^21^

**Figure 3.**
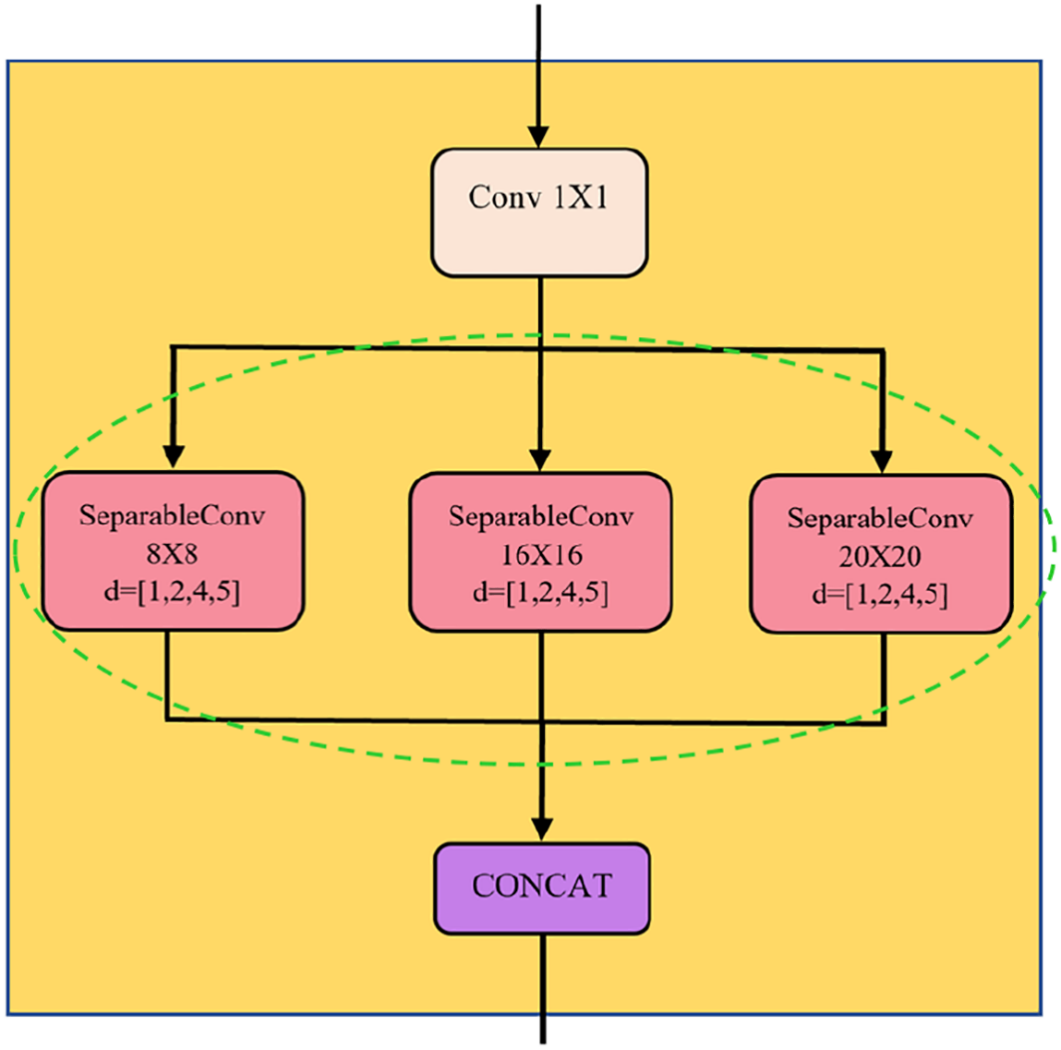
Convolutional unit in MSD block.

**Figure 4.**
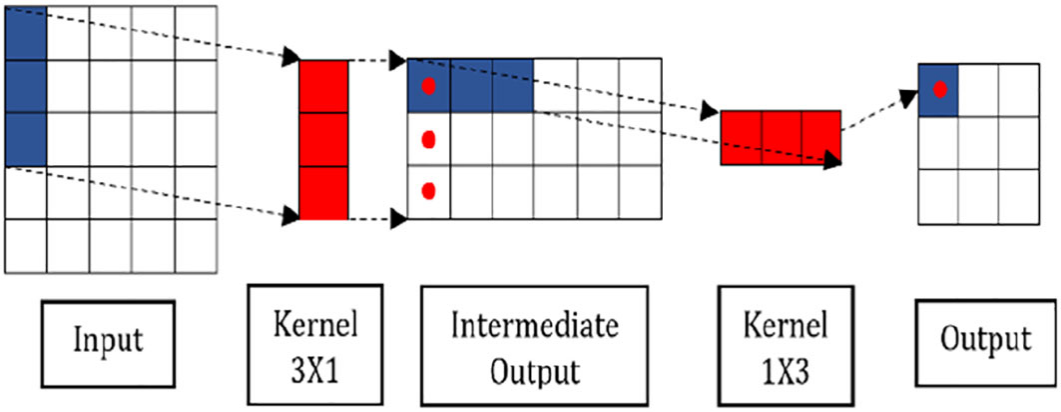
Separable convolution.

Convolutional unit in MSD block extracts spatial-temporal features of the motion data. The components of the convolutional unit are illustrated in Figure 3. First, the input channels are processed via one-by-one causal convolution for dimensionality reduction. This layer, known as bottleneck layer, adopts fewer filters to reduce the number of features maps while the salient features are retained. The causal padding preserves the input sequence's length and order, preventing information leakage from the future into the past. Next, the reduced feature maps are further processed parallelly by separable convolutions (SepConv) with three different-sized filters to extract features at multiple scales. Figure 4 shows the operation of SepConv. The reason for implementing SepConv in MSTCN is that it can produce fewer parameters and reduce computational complexity.

**Figure 5.**
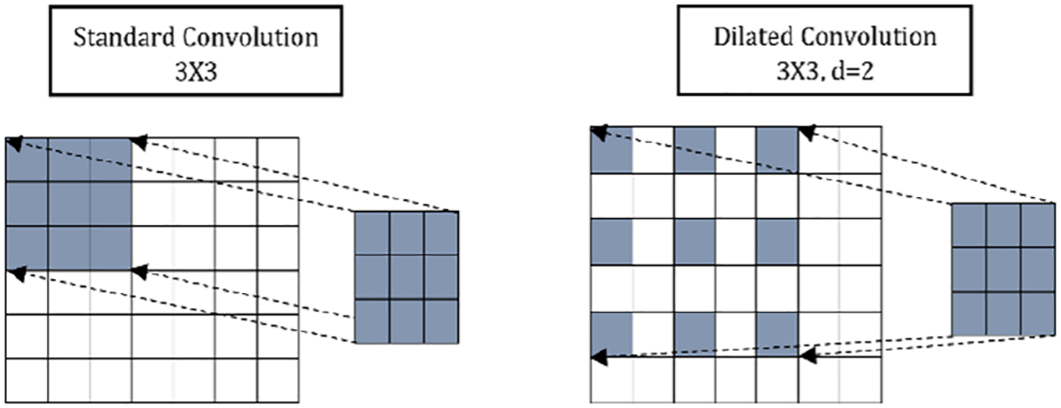
Comparison between normal and dilated convolution.

Dilated convolution prevails over classical convolution because it allows the model to have a larger receptive field, controlled by the dilation rate. This helps capture long-time sequences' global features without increasing the model's parameters and memory. Figure 5 shows the difference between the dilated convolution and the classical/standard convolution. Dilations are implemented in SepConv to increase the receptive fields of the convolution kernels.

The core difference between MSTCN and TCN is that the dilated convolutions are organised parallelly in MSTCN (green dotted circle in Figure 3) but in a serial form in TCN. With the proposed layout, each extracted multiscale feature from the SepConvs with differently sized filters is concatenated for a better model generalisation, see Figure 3.

In a MSD block, average pooling (brown box in Figure 2) down-samples the feature map to reduce noise and dimensionality. Additionally, it also preserves localisation. The pooling's output is fed into a one-by-one convolution. A residual connection is formed by passing the input into a one-by-one convolution, followed by a batch normalisation. This residual connection ensures longer-term dependencies and prevents information loss. Further, it also reduces the vanishing gradient effects. On the other hand, batch normalisations in MSD block are to reduce the internal covariance shift in the model during training. Furthermore, ReLU activation is chosen for its non-linearity, and the gradient vanishing is minimised.

The features extracted from the series of MSD blocks are further fed into the global average pooling (GAP). In MSTCN, GAP replaces the traditional fully connected layers because GAP is more suitable. ^22^ This operation generates one feature map according to each activity from multi-dimensional feature inputs. Besides, GAP is also considered as a structural regulariser since it imposes the generated map as the confidence map for each class. ^22^ With this, it better prevents overfitting by reducing the number of model parameters. Additionally, GAP does not require parameter optimisation.

In the classification stage, a simple softmax classifier is used. The softmax activation formula is defined:

$$\sigma {\left(\vec{z}\right)}_{i}=\frac{e^{z_{i}}}{\sum_{j=1}^{K}e^{z_{j}}}$$ (1)

where $\vec{z}$ is the input vector, $e^{z_{i}}$ is the exponential function of the input, K is the number of classes and $e^{z_{j}}$ is the exponential function of the output. This function outputs probabilities of each class, ranging from zero to one, and the target class will have the highest probability.

### Experimental setup

The experiments were conducted on a desktop with Intel ^®^ Core™ i7-8750H CPU with 2.20 GHz, 16GB RAM and NVIDIA GeForce GTX 1050 Ti with Max-Q Design and 4GB memory. Two public databases, UCI ^12^ and WISDM, ^13^ were used to assess the reliability of the proposed model. In addition, subject independent protocol was implemented where there were no overlapping users between training and testing sets. Details of the databases are recorded in Table 1. The evaluation metrics used in this work include precision, recall, F1 score and classification accuracy.

**Table 1.** Description of UCI and WISDM datasets.

	UCI	WISDM
Sensor	Accelerometer and Gyroscope	Accelerometer
Segment size	128	128
Segment interval	50	20
Channel size	9	3
Training testing split	21 training users: 9 testing users	31 training users: 5 testing users
Validation split	10% of the training set	10% of the training set

### Experiments

Three experiments were conducted on UCI dataset to study the effects of (1) convolution, (2) pooling and (3) regularisation on MSTCN's performance. Table 2 shows the proposed model's performances using dilated one-dimensional (1D) causal convolution (CC) and dilated 1D separable convolution (SC). From the empirical results, it was observed that the parameters of SC are approximately half of the parameters of CC. Usually, models with more parameters perform better since maximal data patterns are captured from the training samples. However, when the training sample size is limited, these models might tend to overfit and not generalise properly to the unseen data, leading to poor performance. In this study, SC obtains ~0.04 higher F1 score than CC.

**Table 2.** Performance of MSTCN using different convolutions.

	Dilated 1D causal convolution	Dilated 1D separable convolution
Number of parameters	6 062 086	3 750 406
Precision	0.9357	**0.9761**
Recall	0.9375	**0.9750**
F1 score	0.9356	**0.9752**
Accuracy	93.62	**97.46**

Next, the performances of max-pooling and average pooling were studied. From Table 3, average pooling dominates max-pooling by attaining F1 score of 0.9752. Average pooling performs better in this domain because it takes every value into account. With this, the information leakage is prevented, and feature localisation is preserved.

**Table 3.** Performance of MSTCN using different pooling layers.

	Max pooling	Average pooling
Precision	0.9478	**0.9761**
Recall	0.9468	**0.9750**
F1 score	0.9463	**0.9752**
Accuracy	94.67	**97.46**

Table 4 shows the performance of MSTCN with different regularisation settings. The regularisation is performed at the bottleneck layer in MSTCN. L1 is good at dealing with outliers and sparse feature spaces. Moreover, it also reduces the coefficient of the insignificant features to zero and removes them. It is a good feature selector. L2 learns complex patterns from the dataset and prevents overfitting. By combining the usage of L1 and L2, we can leverage the benefits from both. Hence, the best result of 97.5% accuracy is obtained with L1 and L2 regularisation.

**Table 4.** Performance of MSTCN using different regularisation settings.

	L1	L2	L1 and L2	Without regularisation
Precision	0.9485	0.9666	**0.9761**	0.9529
Recall	0.9464	0.9650	**0.9750**	0.9521
F1 score	0.9459	0.9649	**0.9752**	0.9517
Accuracy	94.60	96.44	**97.46**	95.28

### Comparison with other state-of-the-art methods

A performance comparison between MSTCN and other state-of-the-art methods was conducted. Tables 5 and 6 show the performance on UCI and WISDM datasets using subject independent protocol. The proposed MSTCN showed extraordinary performances against the existing methods by achieving 97.46% accuracy on UCI and 95.20% on WISDM. The experimental results will be discussed further in the following section.

**Table 5.** Accuracy for user independent UCI dataset.

	Type	Accuracy (%)
Statistical features + SVM ^12^	HCF	96.00
Statistical features + Continuous HMM ^17^	HCF	91.76
Statistical features + HMM Ensemble ^23^	HCF	83.51
Statistical features + RF ^24^	HCF	78.00
Statistical features + Linear SVM ^7^	HCF	86.00
Statistical features + Hierarchical Continuous HMM ^25^	HCF	93.18
Statistical features + Dropout Classifiers ^24^	DL	~76.00
Statistical features + Data Centering + CNN ^26^	DL	**97.63**
CNN ^8^	DL	94.79
Frequency features + CNN ^8^	DL	95.75
Bidirectional LSTM ^10^	DL	93.79
Dilated TCN ^19^	DL	93.80
Encoder-Decoder TCN ^19^	DL	94.60
Statistical features + MLP ^27^	DL	95.00
Frequency and Power features + Multichannel CNN ^28^	DL	95.25
Statistical features + InnoHAR ^29^	DL	94.50
MSTCN (Proposed Method)	DL	**97.46**

**Table 6.** Accuracy for user independent WISDM dataset.

Methods	Type	Accuracy (%)
Statistical features + RF ^24^	HCF	83.46
Statistical features + RF ^6^	HCF	83.35
Statistical features + Dropout Classifiers ^24^	DL	85.36
Statistical features + CNN ^26^	DL	93.32
Dilated and Strided CNN ^9^	DL	88.27
Data Augmentation + Two Stage End-to-End CNN ^18^	DL	84.60
Statistical features + CNN ^30^	DL	94.18
MSTCN (Proposed Method)	DL	**95.20**

## Discussion

From the empirical results, we observe that:

1) MSTCN prevails over HCF methods on both datasets because the proposed model can better capture discriminating features from the motion data. Unlike handcrafted features, these deep features are less biased as they are not dependent on prior knowledge. This is crucial, especially for a subject independent solution.
2) Generally, MSTCN outperforms most CNN-based approaches, with accuracy scores of ~97.5% in UCI and ~95.2% in WISDM. This performance exhibits that MSTCN can capture the global and local features that discriminate each activity. Besides, the implementation of GAP in MSTCN is less prone to overfitting. ^22^ Hence, it is suitable for subject independent HAR.
3) MSTCN dominates the recurrent model ^10^ due to its ability in modelling longer-term dependencies via dilated convolution. Residual connection and ReLU activation in MSTCN allow the model to be less susceptible to gradient vanishing and exploding.
4) MSTCN is a TCN-variant model. The obtained empirical results demonstrate that MSTCN outperforms the ordinary TCNs (Dilated TCN and Encoder-Decoder TCN). ^19^ MSTCN learns features at multiple scales via different convolutions with differently sized filters. The concatenation of these multi-scaled features produces global feature maps encompassing each activity class low-to-high level features, leading to better recognition.

## Conclusions

A new deep analytic model, known as MSTCN, is proposed for subject independent HAR. MSTCN is based on the architectures of the Inception network and temporal convolutional network. In MSTCN, different-sized filters are adopted in dilated separable convolutions to extract multiscale features with the enlarged receptive field of each kernel for longer-term dependencies modelling. Besides, average pooling is performed for dimensionality reduction and locality preservation. The inclusion of residual connections in MSTCN prevents information leakage throughout the network. The efficiency of MSTCN is evaluated using UCI and WISDM datasets. The empirical results demonstrate the superiority of MSTCN over other state-of-the-art solutions by achieving 0.9752 and 0.9470 F1 scores, respectively, in UCI and WISDM.

## Data availability

All data underlying the results are available as part of the article and no additional source data are required.
