# MSTCN: A multiscale temporal convolutional network for user independent human activity recognition

**DOI:** 10.12688/f1000research.73175.2

**Published:** 2022-05-18

**Authors:** Sarmela Raja Sekaran, Ying Han Pang, Goh Fan Ling, Ooi Shih Yin

**Affiliations:** 1 Faculty of Information Science and Technology, Multimedia University, Ayer Keroh, Melaka, 75450, Malaysia; 2 Millapp Sdn Bhd, Bangsar South, Kuala Lumpur, 59200, Malaysia

**Keywords:** human activity recognition, smartphone, temporal convolutional network, dilated convolution, one-dimensional inertial sensor

## Abstract

**Background:** In recent years, human activity recognition (HAR) has been an active research topic due to its widespread application in various fields such as healthcare, sports, patient monitoring, etc. HAR approaches can be categorised as handcrafted feature methods (HCF) and deep learning methods (DL). HCF involves complex data pre-processing and manual feature extraction in which the models may be exposed to high bias and crucial implicit pattern loss. Hence, DL approaches are introduced due to their exceptional recognition performance. Convolutional Neural Network (CNN) extracts spatial features while preserving localisation. However, it hardly captures temporal features. Recurrent Neural Network (RNN) learns temporal features, but it is susceptible to gradient vanishing and suffers from short-term memory problems. Unlike RNN, Long-Short Term Memory network has a relatively longer-term dependency. However, it consumes higher computation and memory because it computes and stores partial results at each level.

**Methods:** This work proposes a novel multiscale temporal convolutional network (MSTCN) based on the Inception model with a temporal convolutional architecture. Unlike HCF methods, MSTCN requires minimal pre-processing and no manual feature engineering. Further, multiple separable convolutions with different-sized kernels are used in MSTCN for multiscale feature extraction. Dilations are applied to each separable convolution to enlarge the receptive fields without increasing the model parameters. Moreover, residual connections are utilised to prevent information loss and gradient vanishing. These features enable MSTCN to possess a longer effective history while maintaining a relatively low in-network computation.

**Results:** The performance of MSTCN is evaluated on UCI and WISDM datasets using a subject independent protocol with no overlapping subjects between the training and testing sets. MSTCN achieves accuracies of 97.42 on UCI and 96.09 on WISDM.

**Conclusion:** The proposed MSTCN dominates the other state-of-the-art methods by acquiring high recognition accuracies without requiring any manual feature engineering.

## Introduction

Human activity recognition (HAR) is extensively applied in various applications such as personal health monitoring, ^1, 2^ geriatric patient monitoring, ^3^ ambient assisted living, ^4^ etc. The widespread use of smartphone-based HAR is due to the ubiquity of smartphones and low-cost sensors. Additionally, sensor-based HAR provides a non-intrusive solution.

Over the years, numerous algorithms have been proposed, including handcrafted feature (HCF) methods ^5- 7^ and deep learning (DL) methods. ^8, 9^ HCF methods require complex signal pre-processing and manual feature engineering to extract essential features. In contrast, DL methods, such as convolutional neural network (CNN), ^8, 9^ recurrent neural network (RNN), and long-short term memory network (LSTM), ^10, 11^ can automatically extract crucial discriminative features from input signals without manual feature engineering. Besides, the architecture is adaptable to different applications.

Though the existing methods produce satisfactory performances, there are several challenges which hinder the HAR models from achieving potential performance:

- HCF methods require manual feature extraction where the extracted features are highly dependent on prior knowledge. This may lead to high bias and missing of essential implicit patterns.
- CNN is good at extracting spatial features. It is suboptimal in learning temporal features. Temporal features are crucial in motion analysis.
- Although recurrent models are feasible for time-series data, RNN is prone to short-term memory problems, leaving out important information at the beginning if the input sequence is too long.
- LSTM prevails over RNN. LSTM has a longer-term dependency and is less susceptible to vanishing gradient. However, LSTM requires higher computation due to multiple gate operations and more memory to store partial results throughout the training phase.

**Figure 1.**
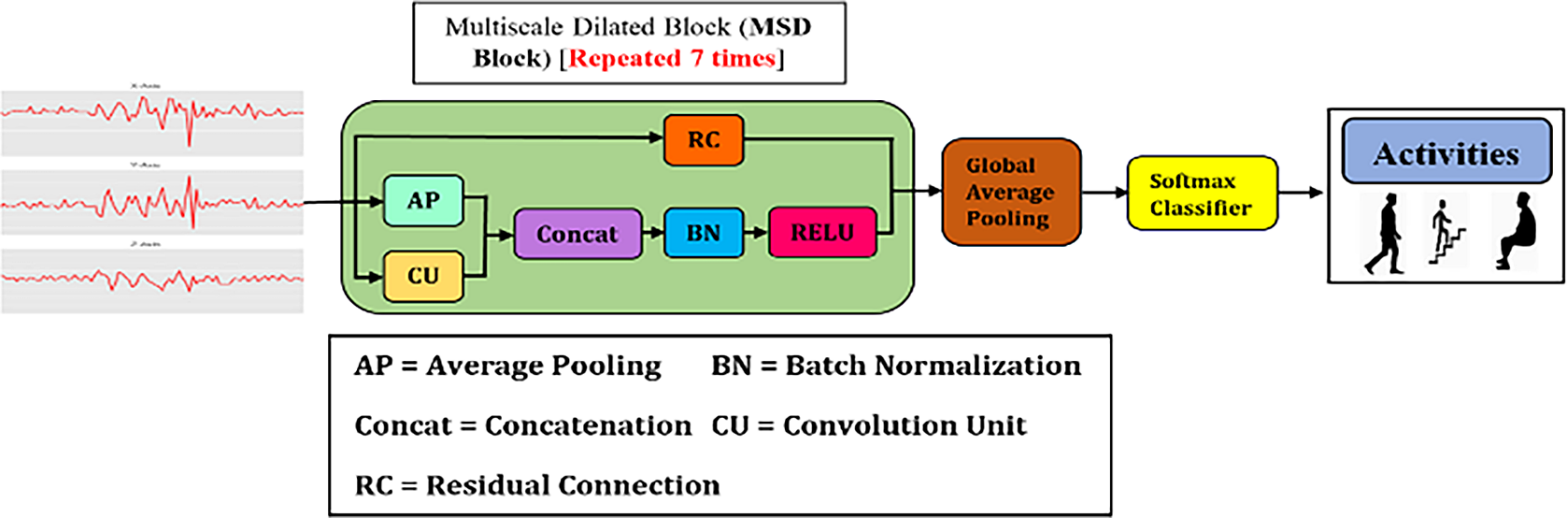
Architecture of MSTCN.

To address the aforementioned challenges, this work proposes a multiscale temporal convolutional network (MSTCN) for HAR. As illustrated in Figure 1, MSTCN is constituted by multiscale dilation (MSD) blocks, global average pooling and softmax. The details of the components will be described in the later section. The contributions of this work are:

- A deep analytic model, amalgamating Inception model and Temporal Convolutional Network (TCN), is developed to extract spatial-temporal features from inertial data. MSTCN requires minimal data pre-processing and no manual feature engineering.
- MSTCN incorporates multiple different-sized convolutions to perform multiscale feature extraction. These multiscale features provide richer information for data analysis.
- To retain longer effective history, dilated convolution is implemented to increase the receptive field without raising the overall parameters.
- A comprehensive experimental analysis is conducted using two popular public databases, UCI ^5^ and WISDM. ^12^ Subject independent protocol is implemented where different subjects are used for training and testing. In other words, there is no overlap in subject in the training and test sets.

### Related work

One-dimensional inertial data undergoes a complicated pre-processing in HCF methods to extract salient statistical feature vectors in time and/or frequency domains. The manually extracted features are then fed into standard machine learning classifiers, such as support vector machine (SVM), ^5, 6^ ADA Boost, ^7^ Random Forest, ^13^ C4.5 decision tree, ^14^ etc., for activity classification. He and Jin ^15^ proposed a discrete cosine transform method to extract features and classify the features using multiclass SVM. Lara *et al*., ^16^ developed an additive logistic regression, boosting with an ensemble of 10 decision stump classifiers. In the works of Ronao and Cho, ^17, 18^ the authors explored the Continuous Hidden Markov Model (HMM) to perform activity recognition in two stages, where the first stage is for static and dynamic classification and the second stage is for course classification. Although these methods produce satisfactory performances, they are highly dependent on the effectiveness of the manual feature engineering techniques.

Recently, researchers leaned towards DL methods since DL requires minimal to zero pre-processing and feature engineering. Ronao *et al*., ^8^ Yazdanbakhsh *et al*., ^9^ and Huang *et al*., ^19^ proposed a CNN-based deep learning system to perform HAR. The reported empirical results show the feasibility of the CNN-based method in analysing motion data. Besides, three-layer LSTM was proposed to classify human activities. ^20^ In addition, Ullah *et al.* proposed a HAR algorithm that classified the normalised inertial data signals using stacked LSTM into respective classes. ^11^ Further, LSTM variant, known as Bidirectional LSTM, was employed in HAR. ^10^ This model uses richer information, i.e. previous and subsequent information, to perform activity recognition. Nair *et al*., proposed two variations of TCN, namely Dilated-TCN and Encoder-Decoder TCN in HAR. ^21^ In addition, another two TCN-based models are proposed in Ref. 22, namely TCN-FullyConnectedNetwork and deepConvTCN. Both works of Nair *et al*., ^21^ and Garcia *et al*., ^22^ concluded that the TCN-based models achieved better performance than existing recurrent models in HAR application due to the longer-term dependencies in TCN.

## Methods

In the proposed HAR, the raw inertial signals were firstly pre-processed to remove noise. Next, the pre-processed signals were segmented using sliding window technique. In specific, the signals were partitioned into fixed-sized time windows and each window did not intersect with another window. Then, the segmented data was fed into MSTCN for feature analysis and classification. MSTCN comprises of MSD blocks (green box in Figure 1), global average pooling and softmax classifier.

**Figure 2.**
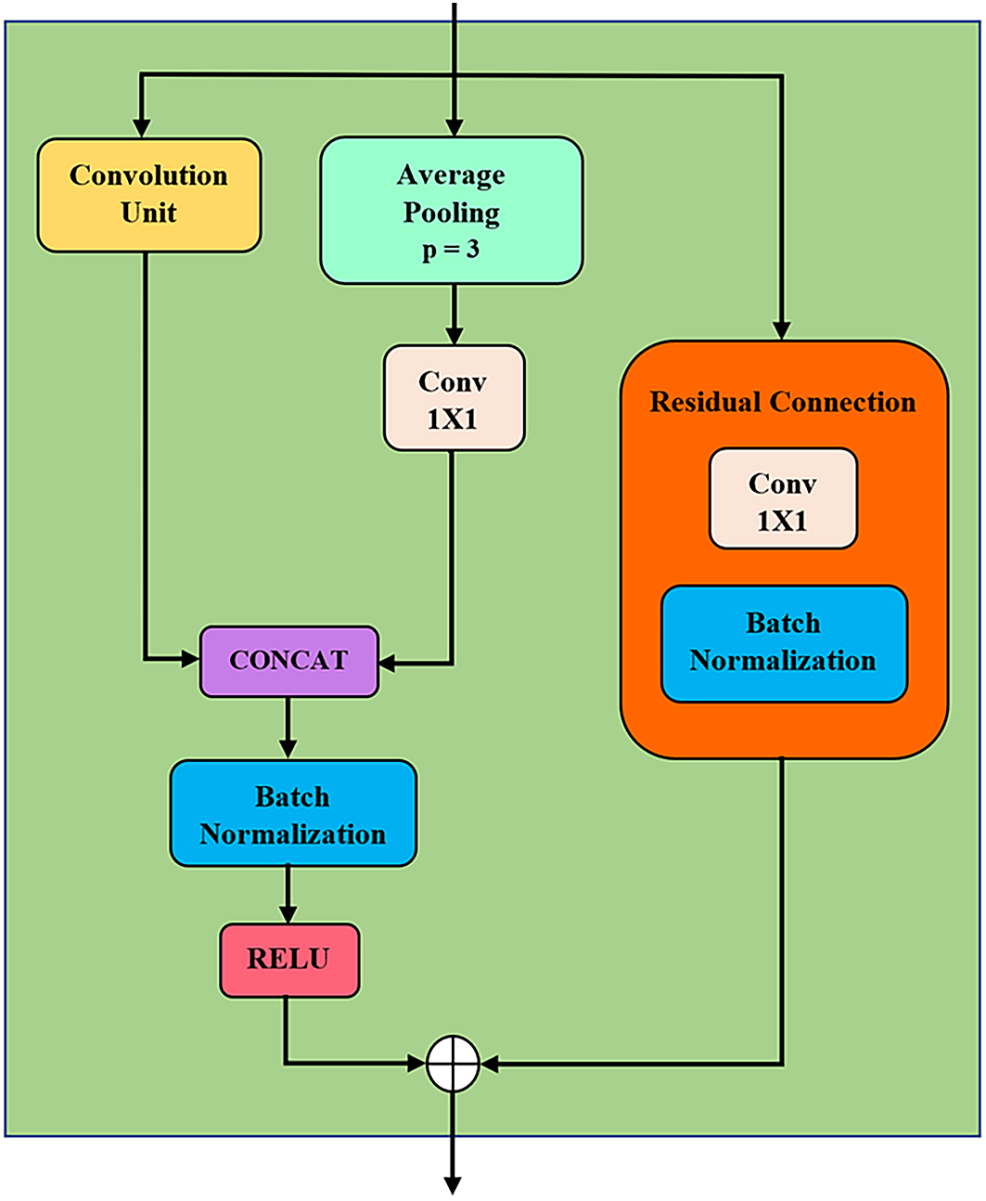
MSD block. (concat = concatenation, conv = convolution and p = pooling factor).

**Figure 3.**
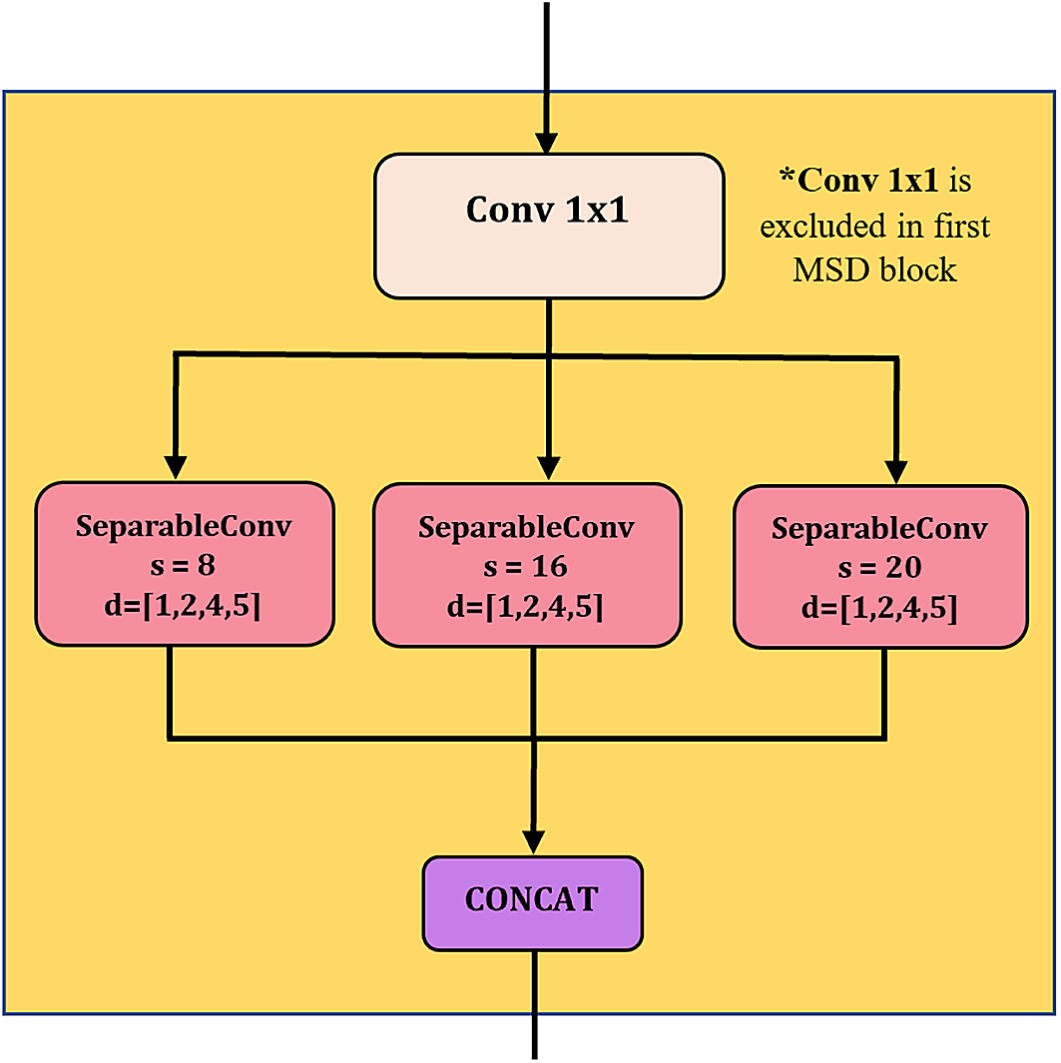
Convolutional unit in MSD block. (concat = concatenation, conv = convolution, s = kernel size and d = dilation rate).

**Figure 4.**
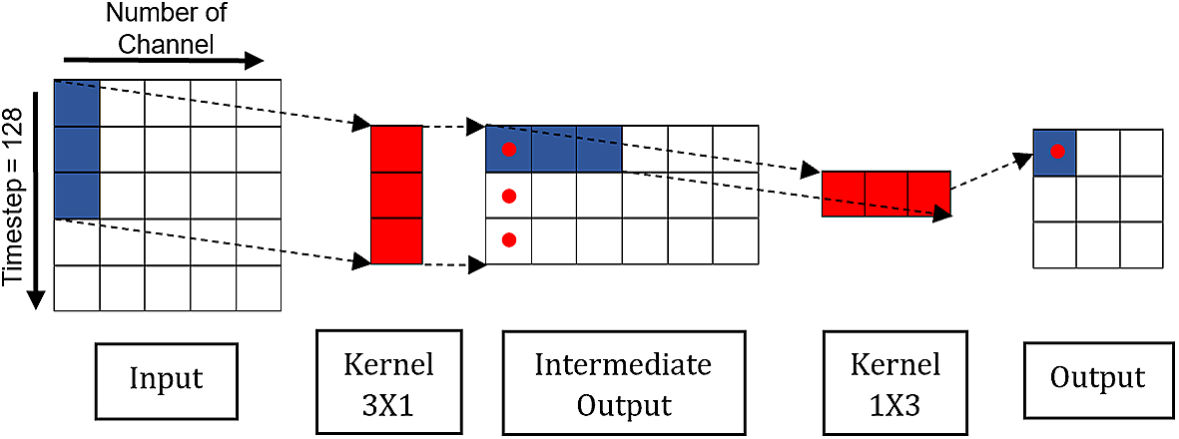
Separable convolution.

Figure 2 illustrates the structure of a MSD block, comprising convolution unit (CU), average pooling, residual connection, batch normalization etc. The design of MSD is inspired by Inception module ^23^ in such a way that multiple kernels/filters are applied simultaneously to the input time series data, as shown in the CU in Figure 3. These kernels are in varying lengths which allow multiscale feature extraction, i.e. extracting features from short and long time series. ^24^ In the subsequent MSD blocks, the input of CU is processed via one-by-one causal convolution for channel-wise pooling and dimensionality reduction. ^25^ The padding preserves the input sequence’s length and order, preventing information leakage from the future into the past. Next, the produced feature maps are further processed parallelly by separable convolutions (SepConv) with three different-sized filters to extract features at multiple scales. The ordinary Inception module is using multiple standard convolutions with smaller kernel sizes, i.e., 3 and 5. ^23^ However, bigger kernel sizes are required in HAR application in order to capture longer time series and preserve longer-term dependencies of the input. ^24^ The authors also claimed that the increasing kernel size leads to the rise of the number of network parameters, which may cause overfitting of the model. Hence, SepConv was used since it reduces the number of parameters in convolution process, while demanding lesser memory compared to standard convolutions. ^26^ Figure 4 shows the operation of SepConv through decoupling standard convolution.

**Figure 5.**
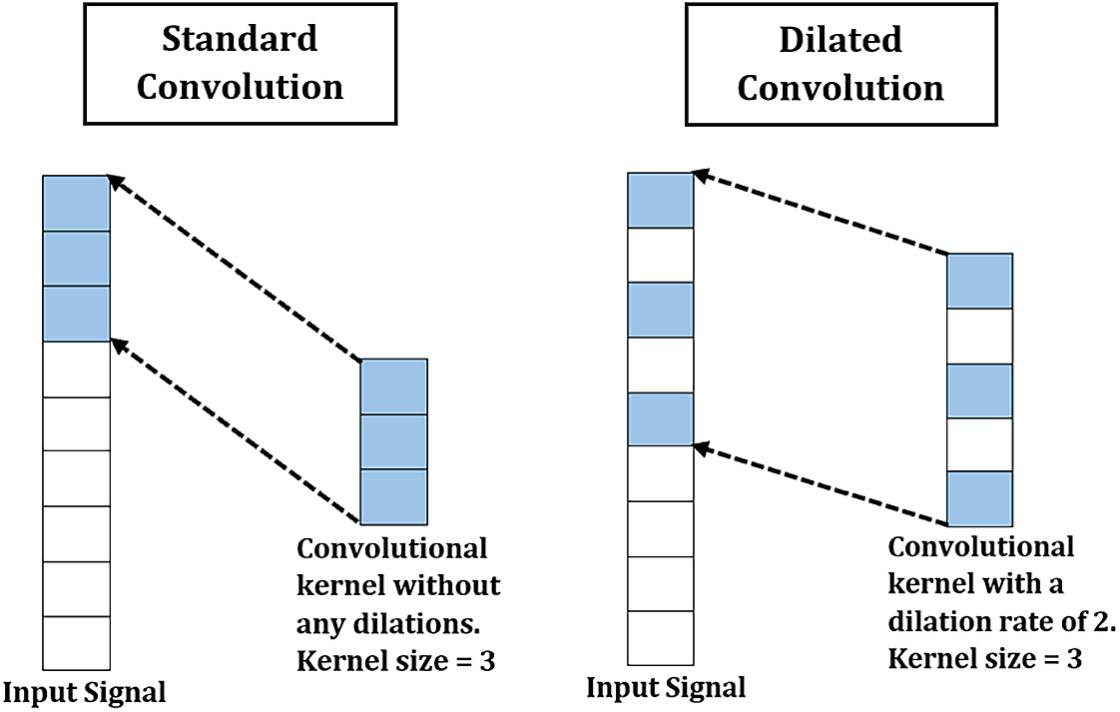
Comparison between standard and dilated convolution.

One of the ways to capture longer time dependent features is by introducing dilations to the convolutions for improving the receptive fields without drastically increasing the model’s parameters. ^27^ The difference between dilated and standard convolution is shown in Figure 5. Receptive field, or field of view, is the region of an input space which is visible to a convolution kernel at a time. A model can capture longer underlying patterns from input data using a convolution kernel with a larger receptive field. The receptive field size of a kernel can be enlarged by increasing the dilation rate. Hence, dilated convolutions were applied in this work to enlarge the receptive field without requiring extra parameters. After the parallel convolutions, the produced feature maps of each SepConv are concatenated by stacking them together, see Figure 3.

In the MSD block, average pooling (in Figure 2) down-samples the feature map to reduce noise and dimensionality. Additionally, it also preserves localisation. The pooling’s output is fed into a one-by-one convolution. Next, the features of CU are stacked with the one-by-one convolution output. As illustrated in Figure 2, a residual connection is formed by passing the input into a one-by-one convolution, followed by a batch normalisation. This residual connection ensures longer-term dependencies and prevents information loss. Further, it also reduces the vanishing gradient effects. On the other hand, batch normalisations in MSD block are to reduce the internal covariate shift in the model during training. Furthermore, ReLU activation is chosen for its non-linearity, and the gradient vanishing is minimised.

The features extracted from the series of MSD blocks are further fed into the global average pooling (GAP) for feature pooling. Next, softmax classifier is implemented for data classification. The softmax activation formula for the *i* ^th^ input vector, σ( *z*) _*i*_, is defined:

$$\sigma {\left(z\right)}_{i}=\frac{e^{z_{i}}}{\sum_{j=1}^{K}e^{z_{j}}}$$ (1)

where *z*_*i*_ is the *i* ^th^ input vector, *e* ^( *z*_*i*_)^ is the exponential function of the *i* ^th^ input vector, *K* is the number of classes and *e* ^( *z*_*j*_)^ is the exponential function of the *j* ^th^ output vector. This function outputs a probability of each human activity class, ranging from zero to one, and the target/predicted class will have the highest probability. Then, softmax loss is computed by implementing categorical cross-entropy loss function to the softmax output.

$${CE}_{\text{general}}=-\sum_{i}^{K}t_{i}\;\text{log}\;z_{i}$$ (2)

where *t*_*i*_ are the ground truths and *z*_*i*_ are the predicted values for *i* ^th^ class in classes *K*

$${CE}_{\text{softmax}}=-\text{log}\;\frac{e^{z_{p}}}{\sum_{j}^{K}e^{z_{j}}}$$ (3)

where *z*_*p*_ is the softmax score for the positive class *p*. The details can be referred to Ref. 28.

### Experiments and results

**Model configuration and experimental setup**

The proposed MSTCN was implemented using Tensorflow, an open-source machine learning platform, with Keras library (a high-level deep learning API written in Python). MSTCN is learned for 100 epochs according to the parameter settings in Table 1. These parameters were fine-tuned based on the validation data from the training set with 10% random data of the training samples.

**Table 1.** Parameter settings of the proposed model.

	UCI	WISDM
Input dimension	(128,9)	(128,3)
Batch size	64	64
Number of MSD blocks	7	7
Number of filters	64	64
Filter size	8, 16 and 20	8, 16 and 20
Dilation rate	1, 2, 4 and 5	1, 2, 4 and 5
Stride	1	1
Regularisation	L1 and L2	L1 and L2
Number of epoch	100	100
Initial learning rate	0.001	0.001
Reduce learning rate on plateau function	Patience: 5 Minimum learning rate: 0.0001 Factor: 0.5 Mode: Validation loss	Patience: 5 Minimum learning rate: 0.0001 Factor: 0.5 Mode: Validation loss
Optimizer	Adam	Adam
Loss function	Categorical cross-entropy	Categorical cross-entropy

The experiments were conducted on a desktop with Intel ^®^ Core™ i7-8750H CPU with 2.20 GHz, 16GB RAM and NVIDIA GeForce GTX 1050 Ti with Max-Q Design and 4GB memory. Two public databases, UCI ^5^ and WISDM ^12^ were used to assess the reliability of the proposed model. In this work, subject independent protocol was implemented to facilitate impersonal solution. There is no overlap in subject between the training and testing sets. This protocol is relatively challenging since there are some extent of discrepancies of gaits towards the motion patterns in same activities. Details of the databases are recorded in Table 2. The evaluation metrics used in this work include precision, recall, F1 score and classification accuracy.

**Table 2.** Description of UCI and WISDM datasets.

	UCI	WISDM
Sensor	Accelerometer and Gyroscope	Accelerometer
Segment size	128 ms ^-2^	128 ms ^-2^
Segment interval	50 ms ^-2^	20 ms ^-2^
Channel size	9	3
Activities (class labels)	Walking, Upstairs, Downstairs, Sitting, Standing and Laying	Walking, Jogging, Upstairs, Downstairs, Sitting and Standing
Training testing split	21 training users: 9 testing users	31 training users: 5 testing users
Validation split	10% of the training set	10% of the training set

### Experiments

Experiments were conducted on UCI dataset to study the effects of (1) convolution, (2) pooling and (3) regularisation on MSTCN's performance. Table 3 shows the proposed model's performances using dilated one-dimensional (1D) causal convolution (CC) and dilated 1D separable convolution (SC). From the empirical results, it was observed that the parameters of SC are approximately half of the parameters of CC. Usually, models with more parameters perform better since maximal data patterns are captured and learned. However, when the training sample size is limited, these models might tend to overfit and not generalise properly to the unseen data, leading to poor performance. In this study, SC obtains ~4% higher accuracy than CC.

**Table 3.** Performance of MSTCN using different convolutions.

	Dilated 1D causal convolution	Dilated 1D separable convolution
Number of parameters	6 062 086	3 750 406
Precision	0.9357	0.9764
Recall	0.9375	0.9744
F1 score	0.9356	0.9747
Accuracy	93.62	97.42

Next, the performances of max-pooling and average pooling were studied. From Table 4, average pooling excels max-pooling with ~3% higher accuracy. Average pooling performs better in this domain because it takes every value into account. With this, the information leakage is prevented, and feature localisation is preserved.

**Table 4.** Performance of MSTCN using different pooling layers.

	Max pooling	Average pooling
Precision	0.9478	0.9764
Recall	0.9468	0.9744
F1 score	0.9463	0.9747
Accuracy	94.67	97.42

Table 5 shows the performance of MSTCN with different regularisation settings. The regularisation is performed at the one-by-one causal convolution in MSTCN. L1 is good at dealing with outliers since it takes the absolute values of all the weight instead of squared value. ^35^ On the other hand, L2 forces weights toward zero, but never exactly zero. The non-sparseness of L2 is useful as a prediction performance. By combining the usage of L1 and L2, we can leverage the benefits of both with achieving ~97.5% accuracy.

**Table 5.** Performance of MSTCN using different regularisation settings.

	L1	L2	L1 and L2	Without regularisation
Precision	0.9485	0.9666	0.9764	0.9529
Recall	0.9464	0.9650	0.9744	0.9521
F1 score	0.9459	0.9649	0.9747	0.9517
Accuracy	94.60	96.44	97.42	95.28

Further, we also conducted the performance comparison between the proposed MSTCN and the other state-of-the-art methods. Tables 6 and 7 records the classification accuracy performance of the methods on UCI and WISDM datasets, respectively.

**Table 6.** Accuracy for user independent UCI dataset.

	Type	Accuracy (%)
Statistical features + SVM ^5^	HCF	96.00
Statistical features + Continuous HMM ^17^	HCF	91.76
Statistical features + HMM Ensemble ^29^	HCF	83.51
Statistical features + RF ^13^	HCF	78.00
Statistical features + Linear SVM ^6^	HCF	86.00
Statistical features + Hierarchical Continuous HMM ^18^	HCF	93.18
Statistical features + Dropout Classifiers ^30^	DL	~76.00
Statistical features + Data Centering + CNN ^31^	DL	97.63
CNN ^8^	DL	94.79
Frequency features + CNN ^8^	DL	95.75
Bidirectional LSTM ^10^	DL	93.79
Dilated TCN ^21^	DL	93.80
Encoder-Decoder TCN ^21^	DL	94.60
Statistical features + MLP ^32^	DL	95.00
Frequency and Power features + Multichannel CNN ^33^	DL	95.25
Statistical features + InnoHAR ^25^	DL	94.50
Stacked LSTM ^11^	DL	93.13
MSTCN (Proposed Method)	DL	97.42

**Table 7.** Accuracy for user independent WISDM dataset.

Methods	Type	Accuracy (%)
Statistical features + RF ^30^	HCF	83.46
Statistical features + RF ^13^	HCF	83.35
Statistical features + Dropout Classifiers ^30^	DL	85.36
Statistical features + CNN ^31^	DL	93.32
Dilated and Strided CNN ^9^	DL	88.27
Data Augmentation + Two Stage End-to-End CNN ^19^	DL	84.60
Statistical features + CNN ^34^	DL	94.18
MSTCN (Proposed Method)	DL	96.09

## Discussion

MSTCN prevails over HCF methods on both datasets because the proposed model can better capture discriminating features from the motion data. Unlike handcrafted features, these deep features are less biased as they are not dependent on prior knowledge. This is crucial, especially for a subject independent solution. Furthermore, MSTCN outperforms most CNN-based approaches, with accuracy scores of ~97% in UCI and ~96% in WISDM. This performance exhibits that the competence of MSTCN in extracting features from the data at assorted scales via the application of different convolutional filter sizes. Besides, GAP in MSTCN not only performing feature pooling, but also minimizes overfitting since there is no parameter to be learned in the GAP. ^36^ This is relatively suitable for subject independent HAR solution since testing data is new/unseen data. Moreover, MSTCN dominates the recurrent model ^10, 11^ due to its ability in modelling longer-term dependencies via dilated convolution. Further, residual connections and ReLU activations in MSTCN allow the model to be less susceptible to gradient vanishing and exploding. MSTCN is a TCN-variant model. The obtained empirical results demonstrate that MSTCN outperforms the ordinary TCNs (Dilated TCN and Encoder-Decoder TCN). ^21^ MSTCN learns features at multiple scales via different convolutions with differently sized filters. These multiscale features provide richer information for data analysis.

## Conclusions

A new deep analytic model, known as MSTCN, is proposed for subject independent HAR. MSTCN is based on the architectures of the Inception network and temporal convolutional network. In MSTCN, different-sized filters are adopted in dilated separable convolutions to extract multiscale features with the enlarged receptive field of each kernel for longer-term dependencies modelling. Besides, average pooling is performed for dimensionality reduction and locality preservation. The inclusion of residual connections in MSTCN prevents information leakage throughout the network. The efficiency of MSTCN is evaluated using UCI and WISDM datasets. The empirical results demonstrate the superiority of MSTCN over other state-of-the-art solutions by achieving ~97% and ~96% accuracy scores, respectively, in UCI and WISDM.

## Data availability

All data underlying the results are available as part of the article and no additional source data are required.
