# Supplementary material for: MSTCN: A multiscale temporal convolutional network for user independent human activity recognition
Source: F1000Res. 2022 May 18;10:1261. Originally published 2021 Dec 8. [Version 2] doi: 10.12688/f1000research.73175.2 (PMC9989544; doi:10.12688/f1000research.73175.2)
Supplement: Peer review history

# Peer review: Reviewer response for version 2

**DOI:** 10.5256/f1000research.133563.r165304

**Published:** 2023-03-06

**Authors:** Xinghua Li

**Affiliations:** 1 School of Remote Sensing and Information Engineering, Wuhan University, Wuhan, China

This work proposed a multiscale temporal convolutional network for human activity recognition. The contribution and innovation are satisfactory. The experiment's result is pleased. This work is meaningful in this field. The authors attempted to polish this manuscript after the first revision.

 It can be indexed as the current version in my opinion.

Is the work clearly and accurately presented and does it cite the current literature?

Yes

If applicable, is the statistical analysis and its interpretation appropriate?

Yes

Are all the source data underlying the results available to ensure full reproducibility?

Yes

Is the study design appropriate and is the work technically sound?

Yes

Are the conclusions drawn adequately supported by the results?

Yes

Are sufficient details of methods and analysis provided to allow replication by others?

Yes

Reviewer Expertise:

Deep learning

I confirm that I have read this submission and believe that I have an appropriate level of expertise to confirm that it is of an acceptable scientific standard.

# Peer review: Reviewer response for version 2

**DOI:** 10.5256/f1000research.133563.r138230

**Published:** 2022-06-13

**Authors:** Cheng-Yaw Low

**Affiliations:** 1 Institute for Basic Science, Seoul, South Korea

The manuscript has been revised accordingly. Particularly, the experimental section has been included with the experimental setup and the important hyper-parameter configurations for reproducibility.

 I have no more comment, and I am happy to recommend this manuscript for indexing.

Is the work clearly and accurately presented and does it cite the current literature?

Partly

If applicable, is the statistical analysis and its interpretation appropriate?

Partly

Are all the source data underlying the results available to ensure full reproducibility?

No

Is the study design appropriate and is the work technically sound?

Partly

Are the conclusions drawn adequately supported by the results?

Partly

Are sufficient details of methods and analysis provided to allow replication by others?

No

Reviewer Expertise:

Deep Learning, Computer Vision, Pattern Recognition, Biometric Recognition.

I confirm that I have read this submission and believe that I have an appropriate level of expertise to confirm that it is of an acceptable scientific standard.

# Peer review: Reviewer response for version 1

**DOI:** 10.5256/f1000research.76807.r102276

**Published:** 2022-02-02

**Authors:** Sultan Daud Khan

**Affiliations:** 1 Department of Computer Science, National University of Technology, Islamabad, Pakistan

In this work, the authors proposed a framework for human activities recognition. The authors proposed a multi-scale temporal convolutional network that constituted multi-scale dilations block to capture multi-scale information. Overall, the paper is not well-written and organized and I have the following concerns the authors need to consider:

1. The contribution of the work is not clear. As there is a lot of literature on human activity recognition systems. How is the proposed framework different from its counterparts?
2. What are the gaps the authors are trying to fill which are left behind by the previous approaches?
3. Figure-1 should be improved, and more details should be incorporated.
4. Discussion section should not be in the bullets form. Please write detail in paragraph.
5. The authors should perform comparison with the following reference:
  - "Stacked lstm network for human activity recognition using smartphone data." In 2019 8th European workshop on visual information processing (EUVIP), pp. 175-180. IEEE, 2019.

Is the work clearly and accurately presented and does it cite the current literature?

No

If applicable, is the statistical analysis and its interpretation appropriate?

Partly

Are all the source data underlying the results available to ensure full reproducibility?

Yes

Is the study design appropriate and is the work technically sound?

Partly

Are the conclusions drawn adequately supported by the results?

No

Are sufficient details of methods and analysis provided to allow replication by others?

No

Reviewer Expertise:

Computer Vision

I confirm that I have read this submission and believe that I have an appropriate level of expertise to confirm that it is of an acceptable scientific standard, however I have significant reservations, as outlined above.

## References

[1] : Stacked Lstm Network for Human Activity Recognition Using Smartphone Data. *2019 8th European Workshop on Visual Information Processing (EUVIP)* .2019; 10.1109/EUVIP47703.2019.8946180 175-180 10.1109/EUVIP47703.2019.8946180

# Response

**Published:** 2022-05-07

**Authors:** Sarmela Raja Sekaran

**Affiliations:** Multimedia University, Malaysia

First of all, we would like to convey our heartfelt thanks to the Editors and Reviewers who have provided us with constructive comments which allowed us to improve our work.

 1. (a) The contribution of the work is not clear. As there is a lot of literature on human activity recognition systems.

**Response:** Thanks for the feedback. The authors have revised the contribution of the work for better clarification. 

 (b)How is the proposed framework different from its counterparts?

**Response:** Thanks for the comment. For better clarification, the authors have revised the part of Contribution in the section Introduction to include the difference between the proposed model and the existing methods:

 Unlike the existing methods, the proposed method does not require either complex signal pre-processing or manual feature engineering by experts. Besides, the proposed MSTCN is capable of extracting features at multiple scales and concatenating them for a better representation of the overall features. Additionally, the adoption of the dilated convolutions enables the proposed model to preserve longer-term dependencies.

 2. What are the gaps the authors are trying to fill which are left behind by the previous approaches?

**Response: **Authors have revised the Introduction section and included the research gap in HAR.

 3. Figure-1 should be improved, and more details should be incorporated.

**Response:** Authors have revised Figure 1 and included more details for better clarification.

 4. Discussion section should not be in the bullets form. Please write detail in paragraph.

**Response:** Authors have revised the Discussion section. The discussion was written in paragraph form.

 5. The authors should perform comparison with the following reference:"Stacked lstm network for human activity recognition using smartphone data." In 2019 8th European workshop on visual information processing (EUVIP), pp. 175-180. IEEE, 2019.

**Response: **Thanks for the suggestion. Authors have included the comparison with the suggested reference in Related Work section and the Experiments section.

# Peer review: Reviewer response for version 1

**DOI:** 10.5256/f1000research.76807.r102273

**Published:** 2022-01-10

**Authors:** Cheng-Yaw Low

**Affiliations:** 1 Institute for Basic Science, Seoul, South Korea

This is a poorly written manuscript, as many sections are unclear and most of the important information is missing.

1. The Inception model is recruited as the network backbone without any justifications. To be specific, why is Inception model useful for HAR? 
2. The methodology section is difficult to read as it is incomplete. For example, the input dimension is unknown? and therefore I do not know  why 1x1 conv is demanded for dimensionality reduction?
  "First, the input channels are processed via one-by-one causal convolution for dimensionality reduction." 
  For clarity, the basic mathematical representation elaborating each operation should be included. On the other hand, I think the methods and results section should be separated into two. 
3. Figure captions contain no detail at all, and this makes the reading very difficult. For example, the authors do not provide in Fig. 3 (and the entire manuscript) the definition for d=[1, 2, 4, 5]? What are represented by 8x8, 16x16, 20x20? How was the feature concatenation is performed? By an arithmetic operation? Or stacking over different feature tensors? 
4. There are a number of ambiguous or misleading statements throughout the manuscript.
  (a)  In MSTCN, GAP replaces the traditional fully connected layers because GAP is more suitable. This operation generates one feature map according to each activity from multi-dimensional feature inputs.
  >>  GAP generates one feature map according to each activity? 
  (b)  First, the input channels are processed via one-by-one causal convolution for dimensionality reduction. This layer, known as bottleneck layer, adopts fewer filters to reduce the number of features maps while the salient features are retained.
  >>  The causal convolutional layer is not a bottleneck layer. 
  (c)  Subject independent protocol is implemented where the training and testing sets do not share the data from the same users.
  >>  The training and the testing sets do not share the data from the same users always. I think the authors are claiming that the training and testing identities (instead of data) are disjoint. State also why this training protocol is important in HAR.
  (d)  L2 learns complex patterns from the dataset and prevents overfitting. 
  >>  L2 is only a normalization technique, and L2 does not learn.
  (e)  The pooling's output is fed into a one-by-one convolution. A residual connection is formed by passing the input into a one-by-one convolution, followed by a batch normalisation.
  >>  There is no residual connection found from both Fig. 2 or 3?
  >>  Dilated convolution captures global features? What are these global features? and by how the global features are captured?
5. This manuscript contains only ONE mathematical equation, but it is problematic. In the meantime, the definitions for each variable should also be provided, e.g., what is z_i? what is meant by "simple" softmax classifier? Cross-entropy?
6. The dataset information, the training procedures and the empirical hyperparameters are not disclosed?
  (a)  What is the input dimension for each dataset? What is the data captured by accelerometer and gyroscope? What are the class number for each dataset?
  (b)  What is feature dimension rendered by the MSTCN? and from which layer the feature representation is extracted for inference purposes? 
  (c)  The optimizer, learning rate, weight decay, batch size, etc., are unknown.
  (d)  The measurement unit for segment size and segment interval in Table 1 should be indicated.

Is the work clearly and accurately presented and does it cite the current literature?

Partly

If applicable, is the statistical analysis and its interpretation appropriate?

Partly

Are all the source data underlying the results available to ensure full reproducibility?

No

Is the study design appropriate and is the work technically sound?

Partly

Are the conclusions drawn adequately supported by the results?

Partly

Are sufficient details of methods and analysis provided to allow replication by others?

No

Reviewer Expertise:

Deep Learning, Computer Vision, Pattern Recognition, Biometric Recognition.

I confirm that I have read this submission and believe that I have an appropriate level of expertise to confirm that it is of an acceptable scientific standard, however I have significant reservations, as outlined above.

# Response

**Published:** 2022-05-07

**Authors:** Sarmela Raja Sekaran

**Affiliations:** Multimedia University, Malaysia

First of all, we would like to convey our heartfelt thanks to the Editors and Reviewers who have provided us with the constructive comments which allowed us to improve our work. 

 1. The Inception model is recruited as the network backbone without any justifications. To be specific, why is the Inception model useful for HAR?

**Response: **Thanks for the feedback. The main reason for recruiting the Inception model as the network backbone is because the Inception model allows multiscale feature extraction. The authors have revised the Methods section to include the justification of recruiting the Inception model for better clarification.

 2. (a) The methodology section is difficult to read as it is incomplete. For example, the input dimension is unknown? and therefore I do not know why 1x1 Conv is demanded dimensionality reduction?

**Response: **The Methods section has been revised for better clarification. In the revised version, the authors have included the input dimension in the Model configuration and experimental setup section. The reason for employing 1x1 Conv has also been included in the Method section.

 (b) I think the methods and results section should be separated into two.

**Response: **Authors have separated the Methods and Results sections as suggested by the reviewer. 

 3. (a)  Figure captions contain no detail at all, and this makes the reading very difficult. For example, the authors do not provide in Fig. 3 (and the entire manuscript) the definition for d=[1, 2, 4, 5]? What is represented by 8x8, 16x16, and 20x20?.

**Response**: Authors have modified Figure 3 and provided a clearer explanation of the figure in the Methods section.

 (b)  How was the feature concatenation is performed? By an arithmetic operation? Or stacking over different feature tensors?

**Response:** Feature concatenation is performed by stacking over different feature maps. The Methods section has been revised to include the information for better clarification.

 4. There are a number of ambiguous or misleading statements throughout the manuscript

 (a)  GAP generates one feature map according to each activity?

**Response:** Authors have revised the explanation in the Methods section for better clarification.

 (b)  The causal convolutional layer is not a bottleneck layer.

**Response:** Authors have revised the explanation in the Methods section for better clarification.

 (c)  The training and the testing sets do not share the data from the same users always. I think the authors are claiming that the training and testing identities (instead of data) are disjoint. State also why this training protocol is important in HAR

**Response:** For better clarification, the section on Model Configuration and Experimental Setup has been revised. The subject independent protocol is adopted in this work. In other words, there are no overlapping users between training and testing sets. This protocol is preferable for real-time HAR applications.

 (d) L2 learns complex patterns from the dataset and prevents overfitting.

**Response:** Authors have revised the explanation in the Experiments section for better clarification.

 (e) There is no residual connection found from both Fig. 2 or 3?

**Response: **Authors have revised Figure 2 and included more information regarding residual connection in the Methods section.

 (f)  Dilated convolution captures global features? What are these global features? and by how the global features are captured?

**Response:** Authors have revised the Methods section for better clarification. Confusing sentences have been revised. Dilated convolution enables longer-term time dependency of the proposed model by enlarging the convolution’s receptive fields.

 5. This manuscript contains only ONE mathematical equation, but it is problematic. In the meantime, the definitions for each variable should also be provided, e.g., what is z_i? what is meant by "simple" softmax classifier? Cross-entropy?

**Response: **Authors have revised and included equations (softmax activation and cross-entropy functions) as well as the definition for each variable of each equation in the Methods section for better clarification. Softmax classifier is implemented in the proposed model for classification purposes. Further, the categorical cross-entropy loss has been implemented to the softmax function output.

 6. The dataset information, the training procedures and the empirical hyperparameters are not disclosed??

 (a) What is the input dimension for each dataset? What is the data captured by the accelerometer and gyroscope? What is the class number for each dataset?

**Response:** Authors have included detailed information about the datasets including input dimension, class number and data type in the Experiments and Results section.

 (b) What is the feature dimension rendered by the MSTCN? and from which layer the feature representation is extracted for inference purposes?

**Response:** Softmax classifier is implemented for inference purposes. The authors have revised the Methods section for better clarification.

 (c) The optimizer, learning rate, weight decay, batch size, etc., are unknown

**Response:** Authors have included information regarding optimizer, learning rate, batch size, etc., in the Model configuration and experimental setup section for better clarification.

 (d) The measurement unit for segment size and segment interval in Table 1 should be indicated.

**Response: **Authors have provided the measurement unit for segment size and segment interval in the Model configuration and experimental setup section.
